# A proposed population-health based metric for evaluating representativeness of air quality monitoring in cities: Using Hong Kong as a demonstration

**DOI:** 10.1371/journal.pone.0252290

**Published:** 2021-05-28

**Authors:** Tilman Leo Hohenberger, Wenwei Che, Jimmy C. H. Fung, Alexis K. H. Lau

**Affiliations:** 1 Division of Environment and Sustainability, The Hong Kong University of Science and Technology, Clear Water Bay, Hong Kong, China; 2 Department of Mathematics, The Hong Kong University of Science & Technology, Clear Water Bay, Hong Kong, China; 3 Department of Civil and Environmental Engineering, The Hong Kong University of Science and Technology, Clear Water Bay, Hong Kong, China; 4 Institute for the Environment, The Hong Kong University of Science & Technology, Clear Water Bay, Hong Kong, China; Northeastern University, CHINA

## Abstract

City air quality monitoring (AQM) network are typically sparsely distributed due to high operation costs. It is of the question of how well it can reflect public health risks to air pollution given the diversity and heterogeneity in pollution, and spatial variations in population density. Combing high-resolution air quality model, spatial population distribution and health risk factors, we proposed a population-health based metric for AQM representativeness. This metric was demonstrated in Hong Kong using hourly modelling data of PM_10_, PM_2.5_, NO_2_ and O_3_ in 2019 with grid cells of 45m * 48m. Individual and total hospital admission risks (%AR) of these pollutants were calculated for each cell, and compared with those calculated at 16 monitoring sites using the similarity frequency (SF) method. AQM Representativeness was evaluated by SF and a population-health based network representation index (PHNI), which is population-weighted SF over the study-domain. The representativeness varies substantially among sites as well as between population- and area-based evaluation methods, reflecting heterogeneity in pollution and population. The current AQM network reflects population health risks well for PM_10_ (PHNI = 0.87) and PM_2.5_ (PHNI = 0.82), but is less able to represent risks for NO_2_ (PHNI = 0.59) and O_3_ (PHNI _=_ 0.78). Strong seasonal variability in PHNI was found for PM, increasing by >11% during autumn and winter compared to summer due to regional transport. NO_2_ is better represented in urban than rural, reflecting the heterogeneity of urban traffic pollution. Combined health risk (%*AR*_*total*_) is well represented by the current AQM network (PHNI = 1), which is more homogenous due to the dominance and anti-correlation of NO_2_ and O_3_ related %AR. The proposed PHNI metric is useful to compare the health risk representativeness of AQM for individual and multiple pollutants and can be used to compare the effectiveness of AQM across cities.

## Introduction

Urban air-pollution is a severe driver of mortality and loss of disability-adjusted life years (DALY), and associated with a number of short -and long-term health complications such as respiratory diseases [1], pulmonary diseases [2], cancer [3] or heart diseases [4]. Globally, more than 80% of city-dwellers experience air quality levels that exceed the World Health Organization’s (WHO’s) limits [5]. Dominant air pollutants include particulate matter of various sizes (generally classified as PM_10_ with a diameter of < 10 micrometres, and PM_2.5_ with a diameter of < 2.5 micrometre), as well as gaseous pollutants as Nitrogen Dioxide (NO_2_) and Ozone (O_3_) [6, 7].

In many cities, air quality measured from fixed-site monitoring (FSM) stations is typically used for public information and regulatory compliance [8]. Due to high expense and complexity in operation, the distribution of FSM is typically sparse in space, e.g. district-based [9]. However, due to complexities in weather, emission and urban morphology, urban air quality exhibit high spatial and temporal variability [10]. Knowing how well FSM can reflect variations in air quality becomes important for epidemiological studies, compliance to regulation, decision making of individual citizens and while setting up, moving or removing obsolete sensors in a network.

Many approaches have been developed to evaluate the representativeness of FSM, including simple geometry [11], classification by land use [12, 13], remote sensing [14], chemical transportation models [15, 16], computational fluid dynamics [17] or classification based on environmental parameters [18]. In recent years, high-resolution air quality data down to street level become available with the development of urban air quality models. For example, Rodriguez *et al*. used the Parallel Micro-Swift-Spray (PMSS) to evaluate the representativeness of FSM, which achieved a resolution of 3 metres [19]. However, this and the previous methods are mostly developed to evaluate spatial representation [20] as well as the ability to detect standard violations [21]. There is a lack of consideration of health impacts, which is the primary goal in formulating air quality guidelines and control strategies. A paradigm of health-based evaluation is needed to judge and compare the representativeness of FSMs to deliver proper information for public health.

The health risk of air pollution at a certain place depends on the abundance of different air pollutants measured or estimated at that location and the toxicity of air pollutants. Concentrations reported by FSM may reflect the abundance of different air pollutants. Still, these concentrations cannot be added or compared among pollutants for health risk because of differences in toxicity. For example, the added short-term health risk in hospital admission is a 0.51% per unit increase in O_3_ concentration (10 μg/m^3^) while it is a 0.28% per unit increase in PM_10_ concentration (10 μg/m^3^) in Hong Kong [22]. To deliver health risks information from total air pollution, Stieb *et al*. proposed an air quality health index (AQHI) which transformed the air pollutants concentrations at a given location into additive health risks [23]. The resulting added health-risks (%*AR*_*total*_) from the AQHI method can be used to express total health risks of air pollution at a given location as well as contributions to it from different pollutants, which can be a useful metric in evaluating the representatives of FSMs for health risks.

Total health impacts of an urban population do not only depend on the geographical variability in health risks calculated by %AR, but also on the population distribution. In a single city, people live in places with substantial variability in air quality. In epidemiological studies, areas with higher population density would be granted heavier weights when estimating the total health impacts for the whole population [24, 25]. Modern cities, such as Hong Kong are very heterogeneous in population distribution, which may lead to substantial differences in total health risk estimates among FSMs compared to traditionally used space-based method. This is especially important when evaluating whether an FSM should be set up, moved or removed from a monitoring network regarding the public health for the whole community.

In the light of the increasing importance of inter-city competition [26, 27], city-networks [28], urbanisation [29], and fast-changing pollution levels [30, 31], a comparable metric to judge the quality of air-pollution management would enormously benefit accountability and good-governance efforts. As urban areas around the globe are facing locally different air-pollution challenges and individual pollutants contribution to total health impacts can vary, such a metric should be based on the representation of public health impacts from air-pollution derived of local risk factors, and not on the representation of individual pollutant species’ concentration levels.

Following these considerations, this study proposes a population-health risk-based method in evaluating the representativeness of urban FSM networks, which is able to: (1) assess and compare the representativeness of health risks among FSMs for different pollutants (including PM_10_, PM_2.5_, NO_2_, and O_3_) and for total short term health impacts (%*AR*_*total*_); (2) evaluate the representativeness of whole air quality monitoring network for single and combined health risks of pollutants.

The method will be demonstrated using Hong Kong as a testbed. There are sixteen FSMs spatially distributed over an area of 1,100 km^2^ in Hong Kong, located in areas of different functionalities such as roadside, residential, commercial, industrial urban areas, and rural areas. The accessibility of air quality monitoring data from these stations provides a unique opportunity to investigate the representativeness of FSMs regarding heterogeneity in their geographic location and function.

## Materials and methods

This section includes: (1) study area and FSMs that would be assessed in this study; (2) a description of high-resolution air quality models, which provide fine-scale air quality data to compare with the air quality reported by FSM; (3) heath-risk based evaluation method for FSM representativeness evaluation; and (4) population-health based network index.

### Study area and FSMs

Hong Kong is selected to demonstrate the proposed metrics due to its heterogeneity in geographical features, population density and land-use, which are common in modern cities.

**Fig 1:**
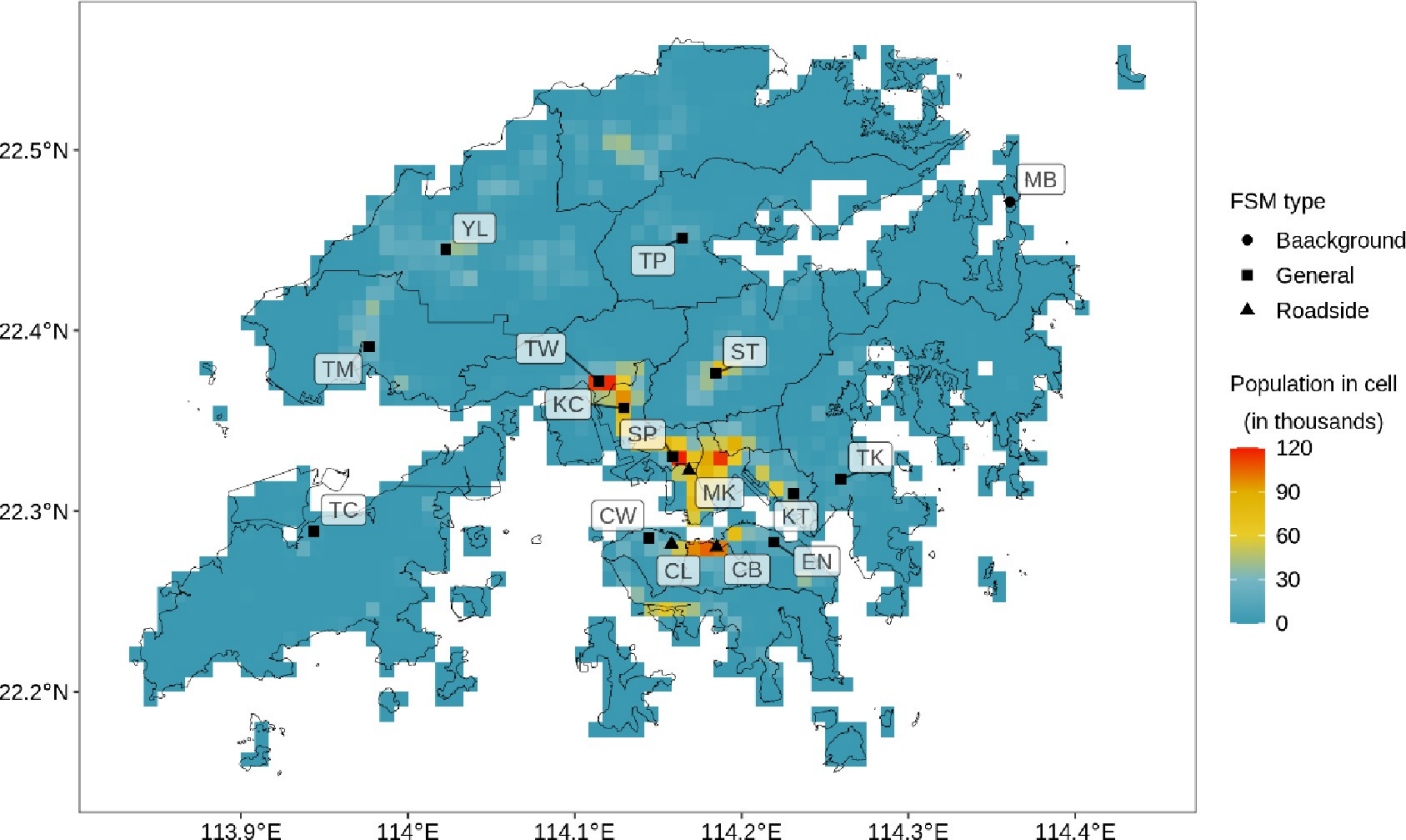
Population density and fixed site monitor (FSM) locations in Hong Kong’s districts, with FSM abbreviated as: Causeway Bay (CB), Central (CL), Central Western (CW). Eastern (EN), Kwai Chung (KC), Kwun Tong (KT), Tap Mun (MB), Mong Kok (MK), Sham Shui Po (SP), Sha Tin (ST), Tung Chung (TC), Tseung Kwan O (TK), Tuen Mun (TM), Tai Po (TP), Tsuen Wan (TW), Yuen Long (YL). Outlines of Hong Kong’s districts reprinted from Esri China (Hong Kong) under a CC BY license, with permission from Esri China (Hong Kong), original copyright 2017.

Hong Kong’s territory is located at the south-eastern tip of China and encompasses 1111 km^2^ of land [32]. The territory can be divided into Hong Kong Island, which on a small slip on its northern coastline is highly urbanized, the highly developed Kowloon Peninsula and the relatively rural New Territories. Densely developed “*New-Towns*” are often central to the commercial and residential life in the New Territories. Due to legislation, only 7% of Hong Kong territory is used for human settlements [32], which makes Hong Kong one of the world’s most densely populated cities. The total population accounts for roughly 7.5 million people [33]. Fig 1 displays the population-distribution in Hong Kong.

Outdoor air pollution has significant seasonal variations in Hong Kong due to the Asian monsoon system (Table 1). The persistent northeast monsoon in winter brings pollution from the Asian continent, whereas the summer monsoon shifts to south-westerly winds that bring in cleaner marine air [34].

**Table 1:** Annual and seasonal means over all general FSM stations for selected air pollutants.

	Unit	Annual	Spring	Summer	Autumn	Winter
**PM**_**10**_	μg/m^3^	32.2	26.7	20.9	40.7	40.6
**PM**_**2.5**_	μg/m^3^	19.2	16.5	12.1	23.9	24.4
**NO**_**2**_	ppb	20.5	19.1	16.8	21.0	25.0
**O**_**3**_	ppb	30.4	29.7	21.1	44.8	26.0

The Hong Kong Environmental Protection Department (HKEPD) runs an FSM network comprising of 12 general stations, three roadside stations and one background station. Locations of FSM stations are shown in Fig 1. Hourly data of PM_10_, PM_2.5_, NO_2_ and O_3_ were obtained from HKEPD for 2019.

General stations are located on rooftops of public infrastructure such as schools, community buildings, or libraries with a height between 13 to 28 metres [35]. The degree of urbanization of surrounding areas varies strongly between stations. However, general stations are typically located in centres of or urban districts and “*New-Towns*” (Fig 1). In contrast, the three roadside stations are located next to busy roads, with inlets on a level of around 3 to 4.5 metres. All three roadside stations are concentrated in the highly developed part of the territory. The background station is located on a small and undeveloped island at Hong Kong’s north-east at 11 metre height. It reflects the background concentration that was transported into Hong Kong in winter due to its upwind location.

### High resolution air quality models

This study takes advantage of a coupled regional and urban modelling system to provide fine-scale air quality data down to street-level. The modelling system consists of a regional model, Community Multiscale Air Quality Modelling System (CMAQ) [36], coupled with an urban transport model, Atmospheric Dispersion Modelling System in the urban area (ADMS-urban) [37].

CMAQ takes inputs from a numerical weather prediction model, Weather Research and Forecasting model (WRF) [38], and a regional emissions model, Sparse Matrix Operator Kernel Emissions (SMOKE) [39], and by taking account of both chemical and physical conversions, produces a regional pollution forecast for nested domains at multiple grid spatial resolution down to 1 km by 1 km. ADMS-Urban then adds spatial resolution for the study area (Hong Kong’s territory) and produces hourly pollution values on a dynamic grid of down to 2-metre resolution by including localized sources (e.g. road-emissions) and urban morphology. A detailed description of the coupled system is available from Che *et al* [10].

Model verification was conducted by comparing model outputs against observational data from FSMs on an hourly basis for the year of 2019. Here, the ADMS-Urban output point closest to an FSM was compared to the actual FSM readings. Following past papers, we used the Index of Agreement (IOA) and Root Mean Square Error (RMSE) as the necessary model verification [10, 13, 40]. Formulas for IOA and RMSE are given in Eqs 1 and 2 [41].

### Eq 1: IOA formula

$$IOA=1-\frac{\sum_{i=1}^{N}{\left(P_{i}-O_{i}\right)}^{2}}{\sum_{i=1}^{N}{\left(|P_{i}+\bar{O}|+|O_{i}-\bar{O}|\right)}^{2}}$$ (1)

### Eq 2: RMSE formula

$$RMSE=\sqrt{\frac{\sum_{i=1}^{N}{\left(P_{i}-O_{i}\right)}^{2}}{N}}$$ (2),

where P are predicted (modelled) values and O are observed values.

IOA is used to evaluate to which extent the deviations of hourly observations averaged over all hours corresponds to the deviations of hourly model predictions averaged over all hours [10]. A detailed discussion on IOA as a metric for air-pollution models is available from Kang *et al*. [42].

**Fig 2:**
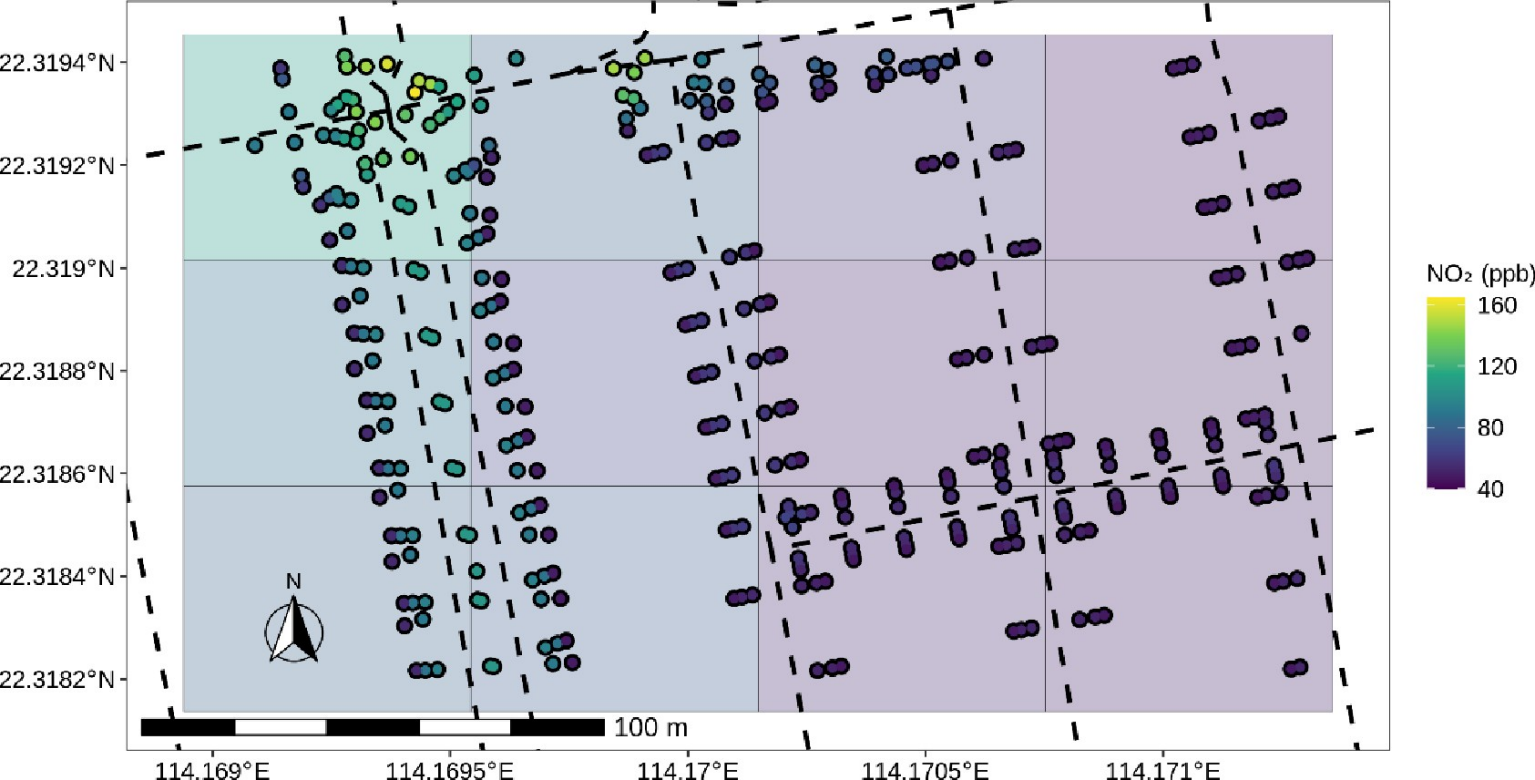
Conversion from dynamic mesh of ADMS model output (points) to raster, with streets (dashed lines).

ADMS-Urban produces a dynamic mesh of output points, with a higher resolution in proximity to pollution sources (roads, industrial sources, etc). To be able to compare areas with a different number of output points and at the same time maintaining a high spatial resolution, we divided Hong Kong’s territory into 1000000 raster cells of each ~3000m^2^ (45 x 48 metres). For every timestep, the concentration-value of a raster cell was set to the arithmetic mean of the concentrations of all ADMS output points inside the respective raster cell. Fig 2 depicts the rasterization of ADMS output points, while at the same time showing the clustering of output points around road sources. The rasterization of the dynamic mesh was achieved using Eq 3.

### Eq 3: Calculation of arithmetic means for all raster cells from model output points

$$\forall i\in \{1,\ldots ,n\}.\;{rc}_{p,i,t}=\frac{\sum_{j=1}^{k_{i}}{mc}_{p,j,t}}{k_{i}}$$ (3)

where, *n* is the number of raster cells, *rc* is the concentration of raster cell for pollutant *p* at timestamp *t*, *mc* is the model output concentration at model output point *j*, and *k* is the number of model output points located in cell *i*.

Further, using a simple intersection between the raster grid and each FSM location, we derived the corresponding raster cell for each FSM station. The pollutant concentrations (after Eq 3) of this corresponding raster cell was set as a “pseudo-station” [19] for each FSM. The representativeness calculation is based on the correlation of these “pseudo-stations” with all other raster cells.

### Health-risk based representativeness evaluation approach

This study introduces the additive health risk approach [23] in developing the metrics for FSM representativeness evaluation, and used local health risks coefficients to calculate the %AR. These coefficients were derived by Wong *et al*. [22, 43] based on the relationship between hospital admissions and measured pollution concentrations in Hong Kong. The hospital admission risks for all age-groups increase 0.022%, 0.028%, 0.045% and 0.051% for every 10*μg*/m^3^ increase in concentrations of PM_2.5_, PM_10_, NO_2_, and O_3_, respectively. These values were used to evaluate the short-term health risks from air pollutants. The long-term health risk is not considered in this study due to a lack of proper health coefficients.

The %AR was calculated for the simulated air pollutants from CMAQ/ADMS models using Eq 4.

### Eq 4: Additional health risk (%AR)

$${\%AR}_{p,j,t}={(e}^{\left(\beta_{p}\times C_{p,j,t}\right)}-1)x\;100\%$$ (4)

Where,

%*AR*_*p*,*j*,*t*_ = added health-risks in the hospitalization of pollutant *p* in cell *j* at timestamp *t* (unitless);

C_*p*,*j*,*t*_ = the modelled pollutant concentration (in μg/m^3^);

*β*_*p*_ = Hospital admission increase rates for pollutant *p*.

A major upside of an %AR based index is its ability to aggregate the health risks of different air-pollutants. The total %*AR*_*total*_ expresses the combined short-term hospitalisation risk from multiple pollutants in a given area (Eq 5).

### Eq 5: Total additional health risk (%*AR*_*total*_)

$${\%AR}_{total,j,t}={\%AR}_{{NO}_{2},j,t}+{\%AR}_{O_{3},j,t}+{max\{\%AR}_{{PM}_{2.5},j,t}+{\%AR}_{{PM}_{10},j,t}\}$$ (5)

Additional health risk of PM takes %AR from either PM_2.5_ or PM_10_, whichever is higher on a given time-step and location (27).

Subsequently, the similarity frequency (SF) method [44] was applied to %AR values (%*AR*_*total*_, as well as individual pollutants) as the basis to calculate health-based representativeness areas. SF aims at calculating the ratio at which a pollutant concentration (may it be measured or modelled) at point A does not differ more than X percent from a pollutant concentration at point B. The methodology of SF has been described before in greater detail [15, 16]. In this work, it is used to compare the similarity in %AR between modelled pollutants at raster cells and modelled pollutants at pseudo-stations. We follow the convention of setting the threshold value for similarity as 20% difference in %AR [13, 19, 44]. SF then equals to the ratio of timesteps that satisfy the similarity criterion against the number of total timesteps. The SF scale ranges from 0 (no representation) to 1 (high representation). A SF > 0.9 shows good representation [44]. We divided the SF scale into further bands, with < 0.5 deemed as low representation, 0.5 < SF < 0.7 as medium representation, 0.7 < SF < 0.9 as medium to high, and SF > 0.9 as high representation. The representativeness of individual FSM was assessed by adding up the total area and population of cells with SF > 0.9.

In the following, Eq 6 was used to derive each cell’s representativeness by the FSM network. For a given cell, the %AR-based SF is calculated between the cell and all pseudo-stations, and the highest value is taken as its representativeness by the FSM network.

### Eq 6: Representativeness of a cell by the FSM network

$$\forall i\in \{PS\}.\;R_{p,j}=max\{{SF}_{{\%AR}_{p,j}\;{\%AR}_{p,i}}\}$$ (6)

Where,

*R*_*p*,*j*_ = health-based representation at raster cell *j*;

*p* = pollutant of pollutant-set, or total pollutants aggregated under Eq 5;

*PS* = cells in which FSM are located (*pseudo-stations*)

${SF}_{{\%AR}_{p,j}\;{\%AR}_{p,i}}$ = SF of %*AR*_*p*, *j*_ (raster-cell) and %*AR*_*p*, *i*_ (*pseudo-station*)

### Population-health network representation index (PHNI)

We defined the representativeness of the FSM network for a given pollutant, or for the sum of the total short-term health effects of all pollutants (%*AR*_*total*_), as the population-weighted health-based representativeness of the target domain, averaged by the total population in the target domain. Eq 7 shows the calculation of the population-health network representation index (PHNI).

The spatial distribution of Hong Kong’s population density was derived from the LandScan dataset [45] with a spatial resolution of 1 km x 1 km, following Lin et al. [46]. The summed population over the study domain is 7.1 million, which is equivalent to the total population of Hong Kong in 2011 [47]. The nearest neighbour algorithm was used to resample the resolution of the LandScan dataset to the resolution of the pollution raster [48]. The target domain can be set to areas of interest, may it be the complete urban territory, or smaller units inside a city (e.g. inner-city administrative boundaries). In the following, we calculated the PHNI for the whole of Hong Kong, as well as for each of the city’s 18 districts, in order to enable relevant inter- and intra-city comparisons.

Seasonal (Summer: June–August, Autumn: September–November, Winter: December–February, Spring: March–May) and annual network indices have been calculated for the year of 2019.

### Eq 7: Population-health network representation index

$${PHNI}_{p,d}=\frac{\sum_{i=1}^{n_{d}}R_{p,i}\times {pop}_{i}}{\sum_{i=1}^{n}{pop}_{i}}$$ (7)

Where, PHNI_*p*,*d*_ is the population-health based network representation index for pollutant *p* in domain *d*, *n*_*d*_ is the number of cells in the target domain *d*, *R*_p,i_ is the health-based representation of cell *i* for pollutant *p* and *pop*_*i*_ is the population count of cell *i*.

## Results

The results include model verification summary, area- and population- based representativeness for aggregated health risks and each selected pollutant, and the annual and seasonal population-health based network indices. We further show the population-health based representation of Hong Kong on the district level.

### CMAQ/ADMS model verification

The coupled CMAQ-ADMS-Urban model performed best at predicting particulates, with an average IOA of 0.7 for PM_2.5_ and of 0.72 for PM_10_ between all stations, based on annual hourly model results and concentration readings (Table 2). The highest IOA was found at TM, a background site close to the neighbouring mainland city of Shenzhen, indicating regional impact on particulates.

**Table 2:** Model result verification based on hourly values of the year 2019 for all FSM stations to their respective pseudo-station (raster-cell) for station-types (B = background, R = roadside, G = general).

Station ID	Station Type	PM_10_		PM_2.5_		NO_2_		O_3_	
		RMSE (μg/m^3^)	IOA	RMSE (μg/m^3^)	IOA	RMSE (ppb)	IOA	RMSE (ppb)	IOA
**MB**	B	16.5	0.74	11.5	0.75	9.1	0.46	0.6	0.54
**CB**	R	24.2	0.66	17.8	0.66	23.6	0.67	0.3	0.32
**CL**	R	21.4	0.67	15.5	0.69	26.9	0.58	0.3	0.36
**MK**	R	20.1	0.74	18.0	0.67	41.2	0.53	0.4	0.52
**CW**	G	18.9	0.74	16.2	0.70	33.1	0.43	0.5	0.65
**EN**	G	18.2	0.74	16.6	0.66	37.7	0.37	0.5	0.64
**KC**	G	16.0	0.74	13.9	0.70	31.9	0.53	0.5	0.57
**KT**	G	21.0	0.69	13.9	0.72	22.1	0.63	0.5	0.55
**ST**	G	17.2	0.71	15.9	0.63	44.0	0.36	0.4	0.64
**SP**	G	18.5	0.73	16.2	0.64	33.8	0.50	0.5	0.60
**TP**	G	19.0	0.72	14.8	0.72	15.8	0.61	0.5	0.53
**TK**	G	15.8	0.75	12.4	0.75	16.8	0.63	0.5	0.57
**TW**	G	18.3	0.72	15.2	0.71	34.8	0.47	0.5	0.57
**TM**	G	25.5	0.69	16.1	0.74	21.8	0.63	0.5	0.56
**TC**	G	20.4	0.68	15.0	0.71	27.1	0.51	0.4	0.58
**YL**	G	23.2	0.72	15.9	0.70	18.8	0.67	0.5	0.53
**Average**		19.6	0.72	15.3	0.70	27.4	0.54	0.5	0.55

The model performed less well for gaseous pollutants (IOA averaged between all stations NO_2_: 0.54; O_3_: 0.55). For NO_2_, the model performed better at roadside stations (mean = 0.59) than general stations (mean = 0.53), indicating the influence of the traffic emission on urban NO_2_ concentrations. For O_3_, the model performed better at general stations (mean = 0.58), and less well at roadside stations (mean = 0.40). This is partly due to the complex interplay of NO_x_ and VOCs concentrations [49] and sunshine [50] acting on O_3_ concentrations at the roadside.

### Spatial map of health risk representativeness

Following our outlined approach, annual and seasonal health-risk representativeness maps for Hong Kong’s territory were generated for %AR of PM_10_, PM_2.5_, NO_2_, O_3_, and combined health risks (%*AR*_*total*_).

**Fig 3:**
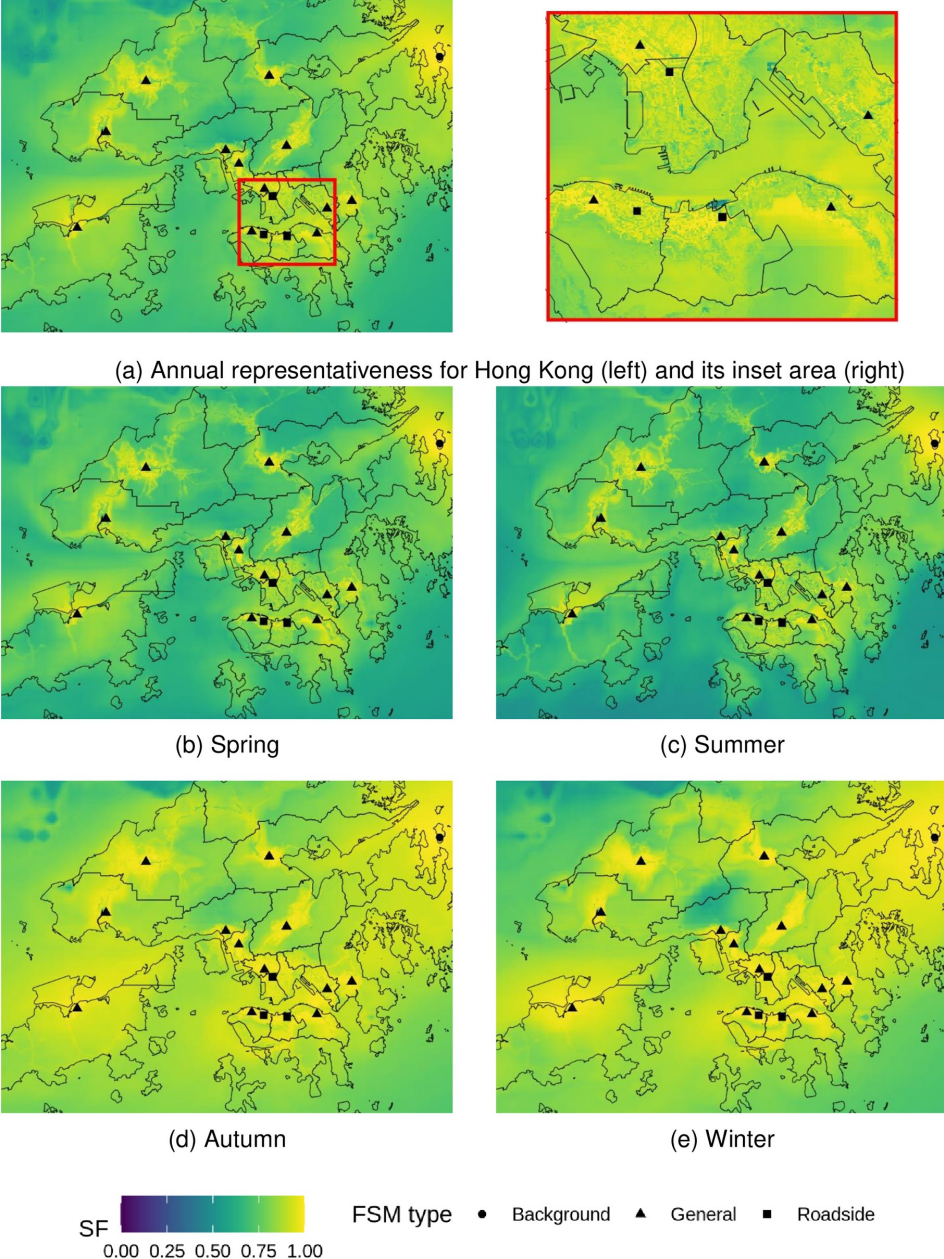
Representativeness of hospital admission risks (%AR) from fixed site monitor (FSM) network over Hong Kong based on annual and seasonal PM_10_ concentrations in 2019. Outlines of Hong Kong’s districts reprinted from Esri China (Hong Kong) under a CC BY license, with permission from Esri China (Hong Kong), original copyright 2017.

**Fig 4:**
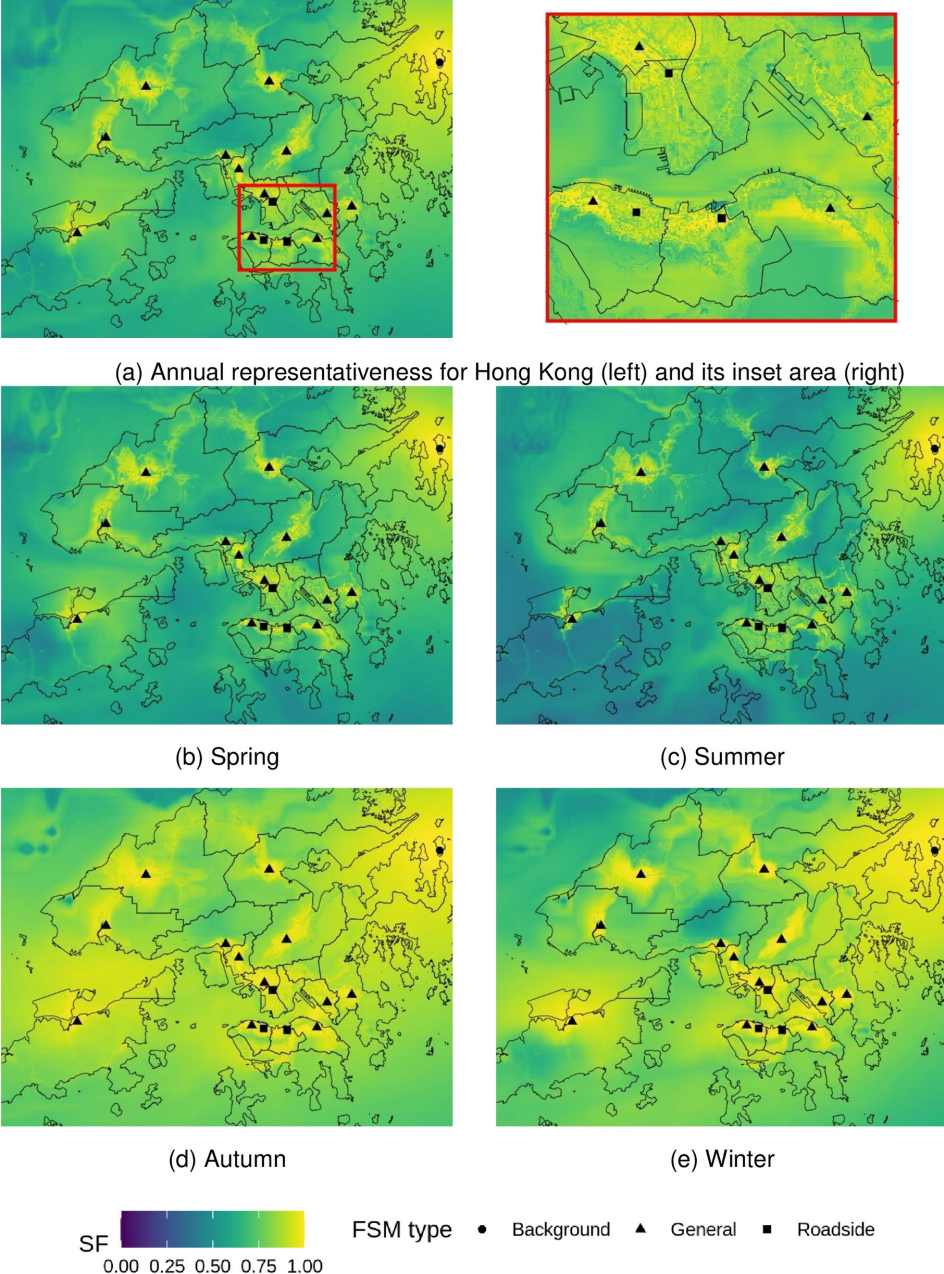
Representativeness of hospital admission risks (%AR) from fixed site monitor (FSM) network over Hong Kong based on annual and seasonal PM_2.5_ concentrations in 2019. Outlines of Hong Kong’s districts reprinted from Esri China (Hong Kong) under a CC BY license, with permission from Esri China (Hong Kong), original copyright 2017.

The current FSM network represents health risks for PM well. Most of the inhabited areas are represented with a SF > 0.9 for PM_10_ (Fig 3) and SF > 0.7 for PM_2.5_ (Fig 4). In Hong Kong, PM concentrations are greatly influenced by regional and super-regional transport [34, 51], accounting for 60–70% of PM_10_ mass [52], and around 40% of PM_2.5_ mass [34]. The good results of PM %AR representation are partially because of the concentration homogeneity resulting from predominantly regional sources, especially for PM_10_. The SF values were stronger in Autumn and Winter due to the enhancement of the regional contribution to PM [52], which leads to elevated concentrations in these two seasons as seen in Table 1. The aggregated areas that were well represented (SF > 0.9) for PM vary substantially among sites. Larger well-represented areas were observed at general stations for PM_10_ (mean = 9.22 km^2^) compared to PM_2.5_ (mean = 4.00 km^2^) than those at road site (PM_10_: mean = 0.99 km^2^; and PM_2.5_: mean = 0.63 km^2^). The sampling inlets for general stations are set well above the ground (13 to 28 metres), which makes them more useful to indicate area-wide air quality, where the sampling heights at the roadside are prone to ground emission (3 to 4.5 meters). The largest well-represented area for PM_10_ (88.39 km^2^) and PM_2.5_ (96.25 km^2^) was found at MB, a background site indicating regional pollution.

**Fig 5:**
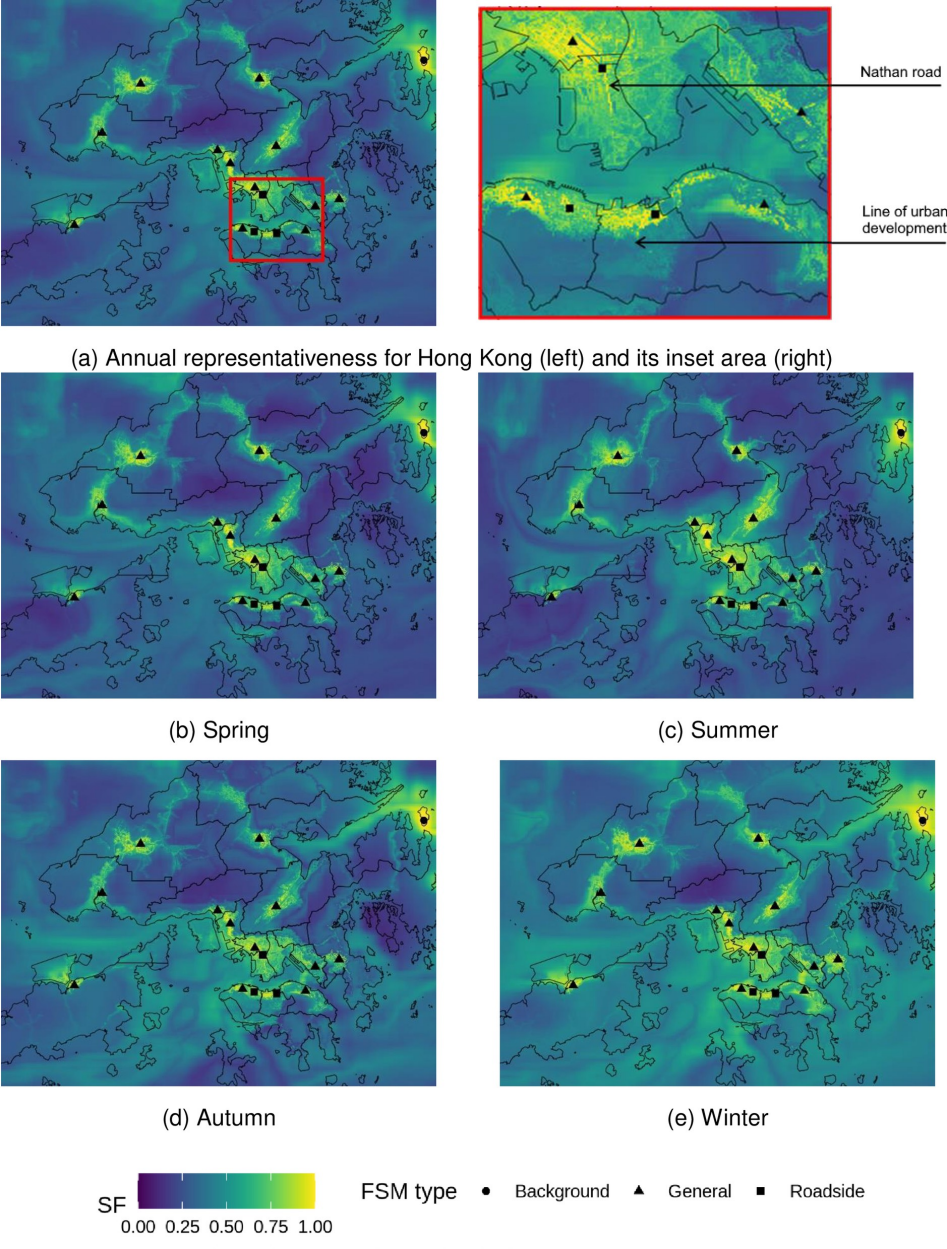
Representativeness of hospital admission risks (%AR) from fixed site monitor (FSM) network over Hong Kong based on annual and seasonal NO_2_ concentrations in 2019. Outlines of Hong Kong’s districts reprinted from Esri China (Hong Kong) under a CC BY license, with permission from Esri China (Hong Kong), original copyright 2017.

The representativeness for health risks related to NO_2_ strongly follows the patterns of urban development, leaving many rural areas underrepresented (Fig 5). There are only 13.4 km^2^ of Hong Kong’s total area is well-represented (SF > 0.9), which are limited to the proximity of stations. This reflects the heterogeneity of NO_2_ pollution which are closely related to urban morphology and local traffic emissions as seen from the inset of Fig 5. For example, clear distinctions in SF values are observed between urban and rural areas in Hong Kong Island. High SF values are concentrated on Nathan road, where a roadside monitor is located with heavy-traffic loads. In rural areas and country parks, the SF values are often lower than 0.3. An exception is the background station, which has approximately 4 km^2^ well-represented (SF > 0.9) areas. This station is surrounded by a large uninhabited area which is less affected by urban morphology and traffic emissions. The spatial representation of %AR NO_2_ patterns are similar throughout the seasons, with slightly higher representation during winter months related to regional pollution.

**Fig 6:**
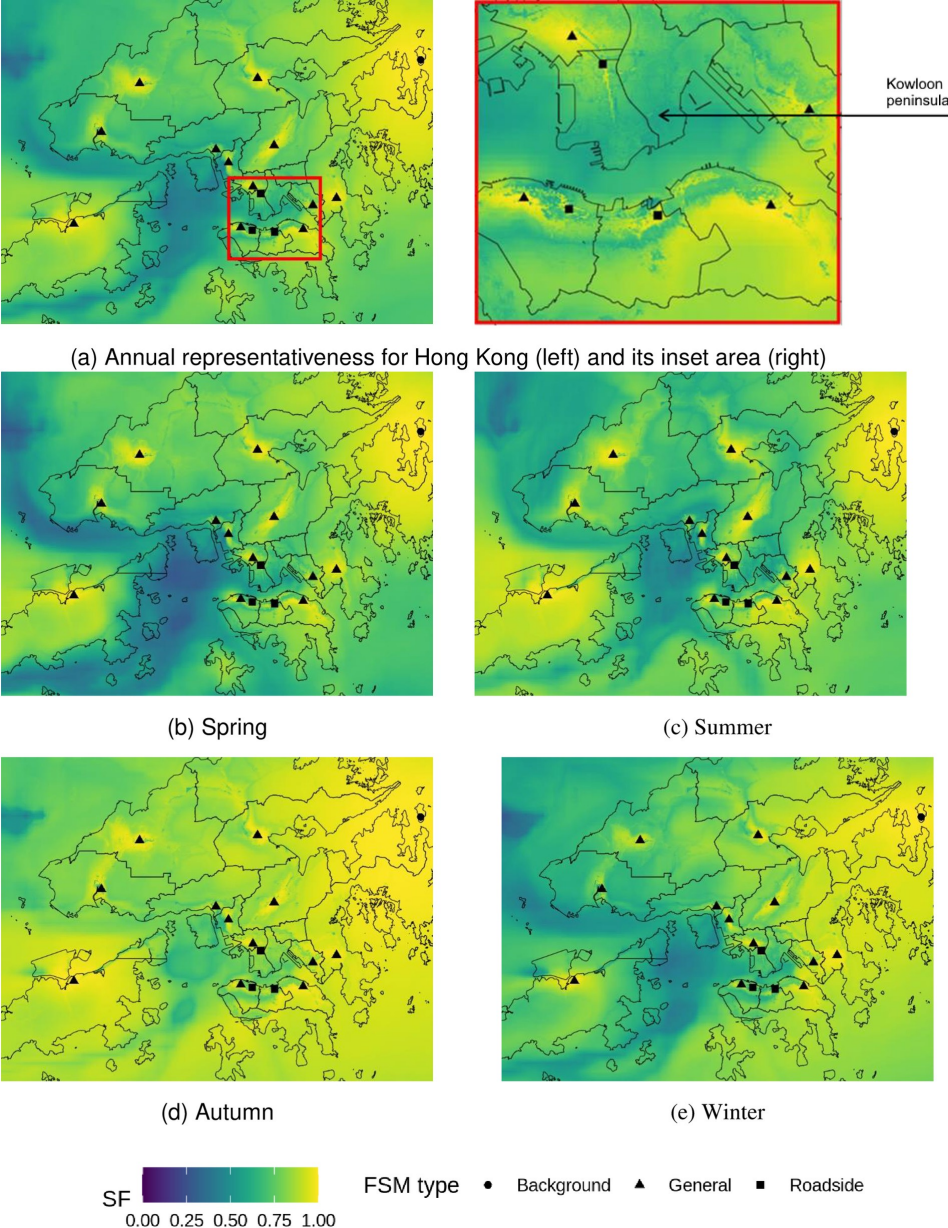
Representativeness of hospital admission risks (%AR) from fixed site monitor (FSM) network over Hong Kong based on annual and seasonal O_3_ concentrations in 2019. Outlines of Hong Kong’s districts reprinted from Esri China (Hong Kong) under a CC BY license, with permission from Esri China (Hong Kong), original copyright 2017.

Most land areas are represented with a SF higher than 0.7 for O_3_ (Fig 6). Compared to NO_2_, there are large rural areas well-represented (SF > 0.9) for health risks related to O_3_ by the current network. For example, the TC station, which is located in a largely undeveloped area, is able to well represent over 20 km^2^ of %AR O_3_. In contrast, the representativeness in some of the developed urban area is poor. For example, the heavily built-up eastern Kowloon peninsula is notably less represented with most SF values smaller than 0.5. Roadside stations contribute little to the spatial representation of O_3_ (mean = 0.19 km^2^ of the area represented with SF > 0.9), which is related to the high heterogeneity in NO_2_ distribution surrounding roads and its titration effect on O_3_. SF values are generally higher in autumn than other seasons, which corresponds to the O_3_ formation peak in Hong Kong and its surrounding regions [53].

**Fig 7:**
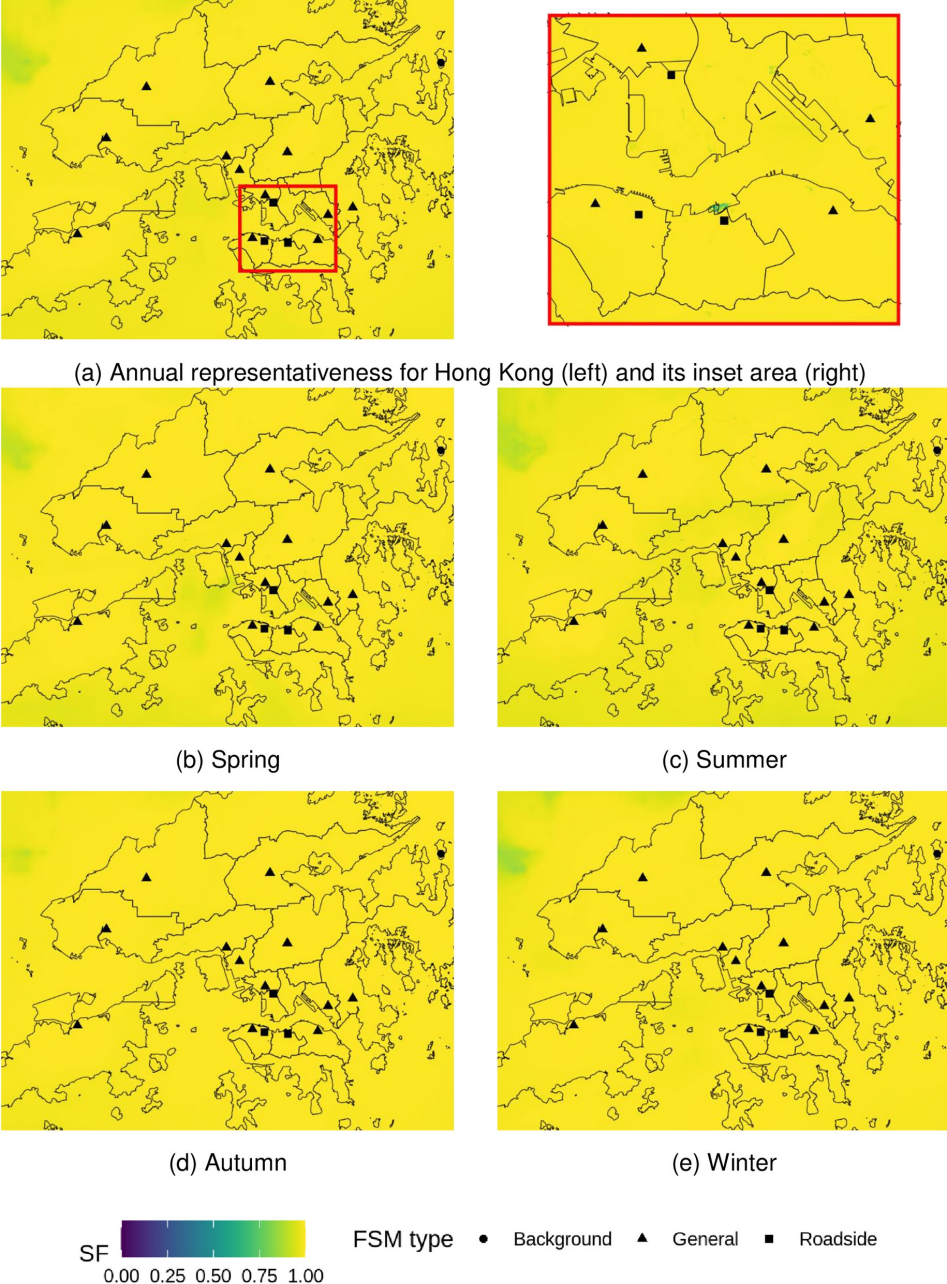
Representativeness of total hospital admission risks (%AR total) from fixed site monitor (FSM) network over Hong Kong in 2019. Outlines of Hong Kong’s districts reprinted from Esri China (Hong Kong) under a CC BY license, with permission from Esri China (Hong Kong), original copyright 2017.

The current FSM network represents the combined health risks (%*AR*_*total*_) well, which is the sum of %AR for all selected pollutants. Almost all land areas are represented with an SF > 0.9, with small exceptions of tunnel exits (Fig 7). This is due to the additive nature of %AR (Eq 5). In Hong Kong, short-term hospital admission risk (%*AR*_*total*_) is highly dominated by NO_2_ and O_3_ (Table 3), which together accounts for approximately 85% to 90% of all short-term hospital admission risks of air-pollution. Although %AR of NO_2_ and O_3_ exhibited substantial variations among FSMs, however, the added health risks are almost homogeneous as listed in Table 3, indicating the homogeneity of oxidative capacity (O_x_) of air pollution throughout the whole domain. Under the influence of sunlight, NO_2_ and O_3_ are chemically converted by a series of reactions without net loss of their combined oxidative capacity [54]. The chemical interplay between NO_2_ and O_3_ leads to anti-correlated patterns for both pollutants [55], but keeps %*AR*_*total*_ values largely constant in space. Due to these effects, the FSM network is achieving good representativeness of %*AR*_*total*_ for both annual and seasons.

**Table 3:** Annual average %AR_total_ at the location of each FSM (pseudo-station), and its contribution from different pollutants.

Station	Station Type	%*AR*_*total*_	Contribution from each pollutant (in %)		
			PM	NO_2_	O_3_
**MB**	Background	5.8	10.7	9.3	80.0
**CB**	Roadside	6.4	15.2	44.5	40.3
**CL**	Roadside	7.1	17.2	56.6	26.2
**MK**	Roadside	6.9	16.7	55.1	28.2
**CW**	General	6.0	13.4	38.1	48.4
**EN**	General	5.8	11.6	19.9	68.5
**KC**	General	5.9	12.9	42.6	44.5
**KT**	General	6.3	14.5	39.0	46.6
**ST**	General	5.9	12.6	34.7	52.7
**SP**	General	6.0	13.7	43.0	43.3
**TP**	General	6.0	12.1	26.9	61.0
**TK**	General	5.9	12.2	22.2	65.7
**TW**	General	5.9	12.7	39.9	47.4
**TM**	General	5.9	12.0	33.7	54.3
**TC**	General	5.8	10.7	19.4	69.9
**YL**	General	5.9	12.1	30.4	57.5

### Population-based health risk representativeness

**Fig 8:**
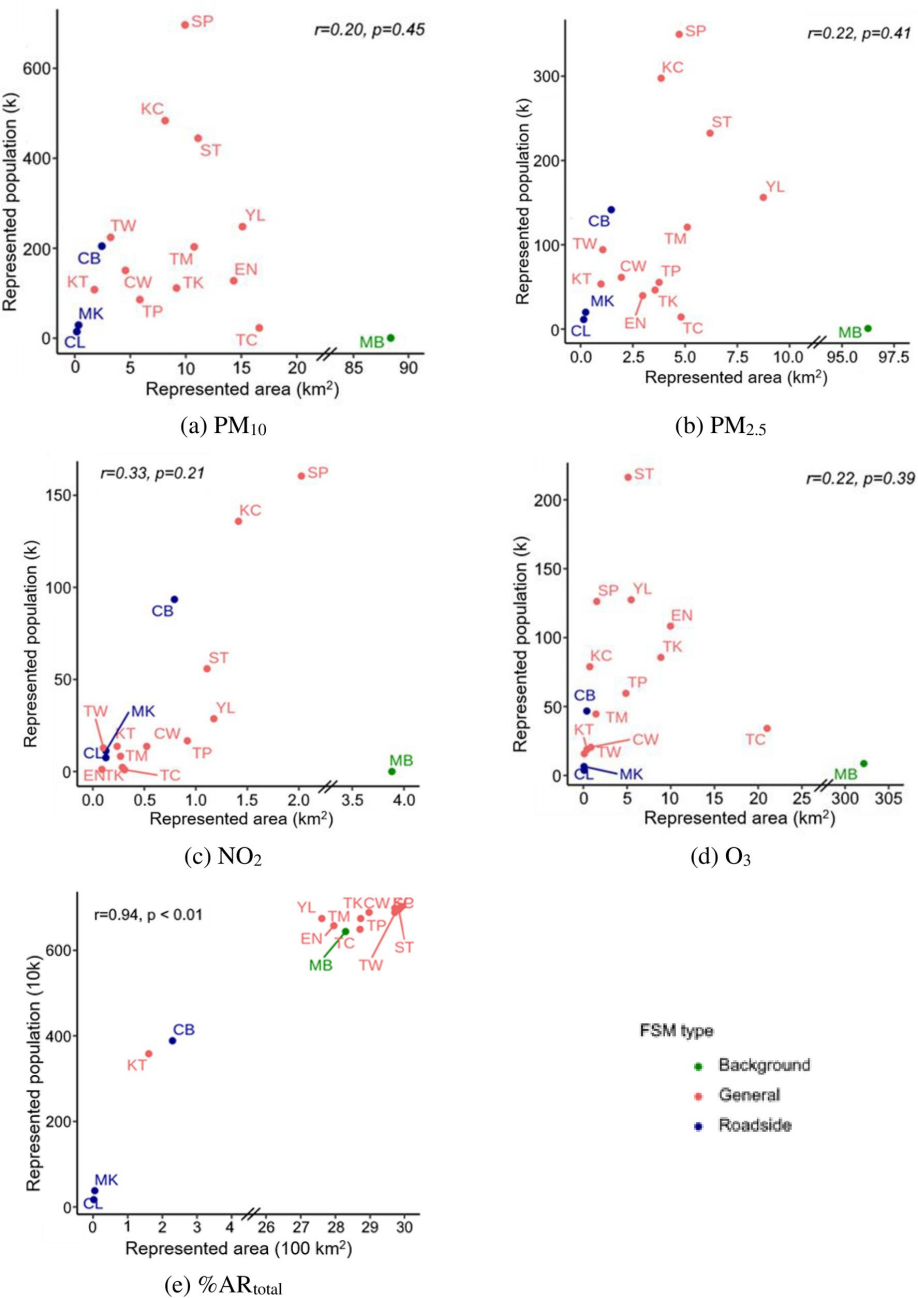
Represented area and represented population (SF > 0.9) by fixed site monitor (FSM).

The aggregate population under well-represented (SF> 0.9) area was 2.7 million, 1.6 million, 1.0 million, and 0.5 million for PM_10_, PM_2.5_, O_3_, NO_2_, respectively, which account for 38%, 23%, 14% and 8% of the total population. The population-based health risk representativeness presents quite different patterns among FSMs compared to the area-based representativeness, as shown in Fig 8. For example, the SP station presents the largest well-represented population for PM, but it only ranks the 6^th^ and 7^th^ in representing areas for PM_2.5_ and PM_10_, respectively. The background station has the largest well-represented areas for all selected pollutants, but least well-represented population among all FSMs. The discrepancies in the ranking between area-based and population-based representativeness reflect the heterogeneity of population distribution. For example, the SP station is located at a densely populated district with average population density of over 40,000 persons/km^2^ while the background station is located on a rural island which barely have no inhabitants.

The relationship between area-based and population-based representativeness is further evaluated using person correlation coefficients. Among the 16 FSMs, the correlations between these two methods are low (r < 0.3) and insignificant for all selected pollutants. Considering that MB station is a background station, which has quite a unique purpose and function than other stations, we re-conducted the correlation analysis by excluding the MB station. Among the 12 general and 3 roadside stations (n = 15), the correlation between well-represented area and well-represented population becomes significant only for NO_2_ (r = 0.86), and remains insignificant for other pollutants. This indicates that the conventional evaluation methods, which are solely based on area is inadequate to address the representativeness of FSMs for public health, especially in the context of high population heterogeneity in urban areas. Although a high correlation is observed for NO_2_, the overall well-represented population is small, less than 10% of the population.

The entire population (99.9%) is well-represented (SF > 0.9) by the FSMs for the combined health risks %*AR*_*total*_. The correlation between well-represented area and well-represented population is high and significant (r = 0.94, p < 0.01). There is a large overlap between FSMs in their well-represented area or population for %*AR*_*total*_, which makes it less sensitive to the heterogeneity in the population.

### Annual and seasonal PHNI

The annual and seasonal PHNI over the entire domain is presented in Table 4 for individual and combined %AR. The PHNI provides an overall evaluation of the representativeness of the FSM network for public health, with values from 0 to 1 representing bad to good quality.

**Table 4:** Annual and seasonal health-based index of representation quality (PHNI) of the FSM network.

Base pollutant	Annual	Spring	Summer	Autumn	Winter
**%AR**_**PM10**_	0.87	0.84	0.83	0.93	0.93
**%AR**_**PM2.5**_	0.82	0.79	0.77	0.92	0.91
**%AR**_**NO2**_	0.59	0.59	0.63	0.60	0.65
**%AR**_**O3**_	0.78	0.77	0.77	0.87	0.79
**%AR**_**total**_	1.00	1.00	1.00	1.00	1.00

Amongst the four pollutants, the FSM network is best able to represent the population’s health risks for PM, with annual PHNI of 0.87 for PM_10_ and 0.82 for PM_2.5_, respectively. Inter-seasonal variation in PHNI is 0.14 (0.77–0.91) for PM_2.5_ and 0.1 (0.83–0.93) for PM_10_, with the highest PHNI found in winter for both PM, indicating a strong regional impacts on public health risks to PM in Hong Kong.

The annual PHNI for NO_2_ is 0.59, which is lower compared to PM. This is major due to the heterogeneity of NO_2_ distributions over Hong Kong which is related to traffic emissions and urban morphology such as street canyons [10]. The inter-season variations in PHNI are relatively small, ranging from 0.59 in spring to 0.65 in winter (Table 4). Under the Asian monsoon system, the air plume in Hong Kong shifts from the south in summer, which will bring fresh ocean air, to North in winter, which will bring continental air pollution from Pearl River Delta region. However, the PHNI for NO_2_ is quite similar between summer (0.63) and winter (0.65), indicating that NO_2_ pollution in Hong Kong is more related to local sources.

The annual PHNI for O_3_ is considerably higher compared to NO_2_, with an annual average of PHNI of 0.78. The PHNI for O_3_ is much higher in autumn (0.87) than other seasons (0.77–0.79), which is related to increase contribution from regional pollution and increased photochemistry under sunlight in autumn.

The PHNI for %*AR*_*total*_ is nearly 1 for both annual and seasonal scales, indicating the current FSM network has a good capacity to represent short-term public health risks to air pollution in Hong Kong. As mentioned before, the short-term risks (%*AR*_*total*_) is dominated by %AR NO_2_ and %AR O_3_, which are anti-correlated in the study domain. The homogeneity nature of the %*AR*_*total*_ makes it less sensitive to the location of FSMs and heterogeneity of the population.

### PHNI comparison among districts

**Fig 9:**
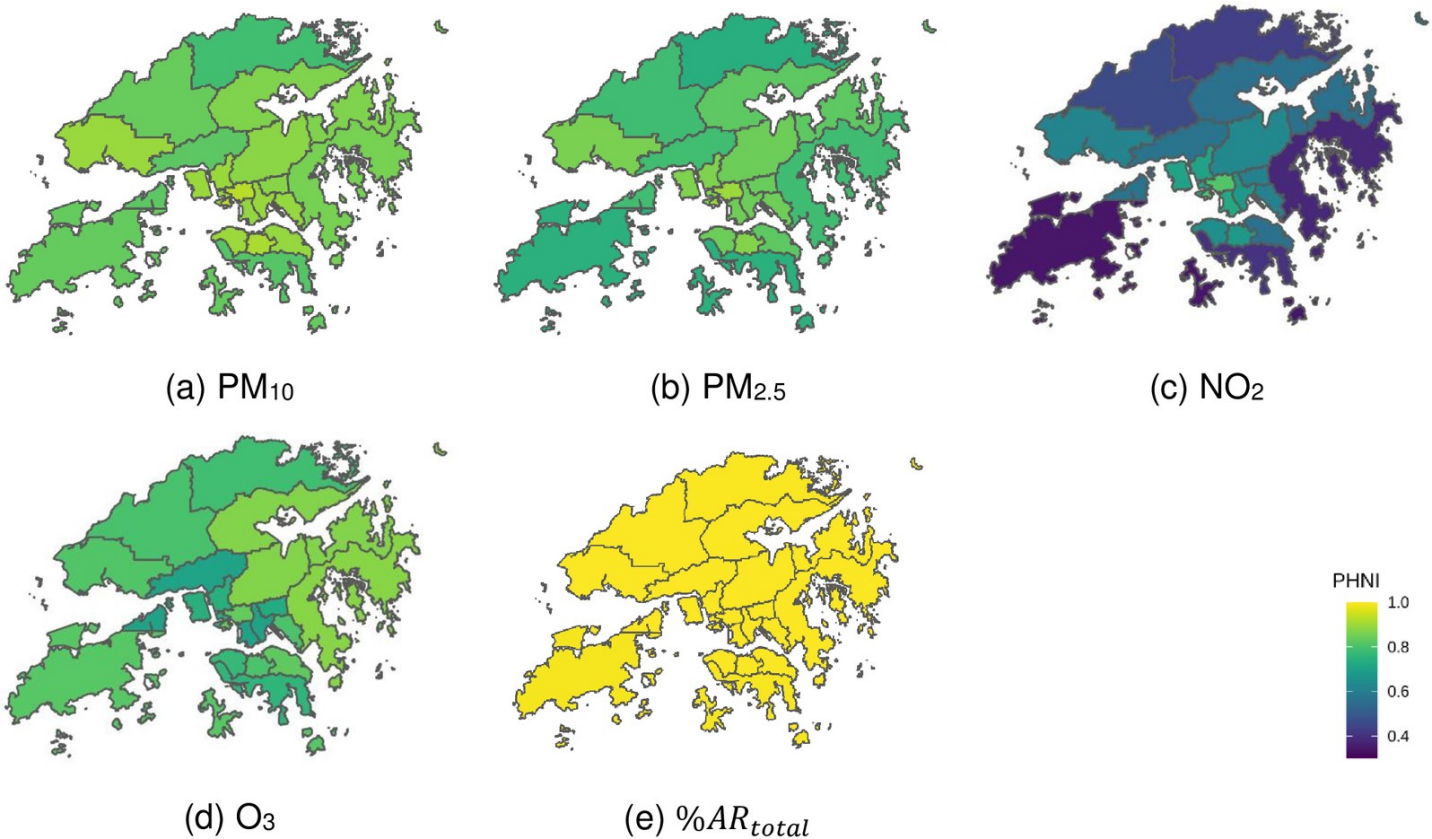
District-level population-based health representativeness for Hong Kong. Outlines of Hong Kong’s districts reprinted from Esri China (Hong Kong) under a CC BY license, with permission from Esri China (Hong Kong), original copyright 2017.

Hong Kong’s territory is divided into 18 administrative districts. Smaller districts are located in the densely populated city centre, and larger districts are predominantly located in the outlying and more rural areas. Annual PHNI was calculated for each district and %AR, as seen in Fig 9.

The district-level PHNI values ranged from 0.74 to 0.9 for PM_2.5_ and from 0.78 to 0.92 for PM_10_. Higher PHNI values were found in inner districts surrounding city centre, such as Sham Shui Po, and lower PHNI values were found in outlying districts such as North and Southern districts. The allocations of existing FSMs are sparser in remote areas than city centres, making it more challenging to reflect the public health risks given the heterogeneity in pollution and population.

The district-level PHNI values ranged from 0.34 to 0.81 for NO_2_. Higher PHNIs are highly concentrated in districts where roadside stations are located. As discussed in previous sections, NO_2_ pollution is closely related to traffic emission. Roadside stations are situated in busy traffic roads with sampling inlet much closer to the ground than general and background sites, which make them more appropriate to reflect public health risks to NO_2_ in their located districts.

The district-level PHNI ranged from 0.71 to 0.88 for O_3_. Higher PHNIs are found in districts near the background station, indicating impacts of background or regional O_3_ pollution on public health risk.

Hospital admission risks of all pollutants combined (%*AR*_*total*_) are well-represented by the current sensor network in all districts (PNHI > 0.99), resulting in a uniform high representation that contrasts the variations found for individual pollutants (Fig 9).

## Discussion

This proposed population-based representation evaluation method is different from existing methods that rely on spatial concentrations [19, 21, 56]. Based on our results, there is no apparent correlation between the size of the represented area and the actually represented population among the FSMs for any of the selected pollutants. Therefore, it cannot be taken for granted that an FSM network successful in spatially representing an urban area is also successful in conveying its population. The proposed population-health based metric integrates population distribution and risk factors with spatial pollutant concentrations in evaluating the representativeness quality of an FSM network, which can better address the concerns on public health risks to air pollution.

Compared to concentration representativeness based on single pollutants, a major advantage of health-based representativeness is its ability to combine the health risks of various pollutants into a single metric (Eq 5). We found high %*AR*_*total*_ representation throughout the whole study area. These results indicate that the current FSM network in Hong Kong can reflect the combined effects of air pollution on short-term health risks well. Nevertheless, it should be noted that the short term %*AR*_*total*_ is more uniformly distributed in Hong Kong due to the dominance and anti-correlation between NO_2_ and O_3_ under the VOC-limited regime. The homogeneity nature of %*AR*_*total*_ may not hold in other places and thus it should not be taken for granted that the FSM network will always reflect well the combined effects.

The proposed PHNI index is not only useful to evaluate the quality of an existing FSM network but also can help improve the network during design or in operation. A network with much overlap is unnecessarily expensive, and a cost-effective solution always aims at reducing station overlap [21, 57]. An optimization effort aimed at maximising PHNI removes the problem of overlap, as it is aimed at optimizing multi-pollutant based %*AR*_*total*_, with only the best representation by any FSM station of a given cell counting into the metric (Eq 6). Therefore, the decision of whether and where to add or remove an FSM sensor should always be driven from a holistic network-perspective in the interface of existing stations, population exposure and the health effects of the pollutant.

The %AR for each individual pollutant is also important for provide health risk information. Some subgroups of the population are especially vulnerable a single pollutant, e.g. through an allergic pathway [58, 59]. For these groups, the representation of a specific pollutant is critical. Besides, current metrics are not integrating long-term health risks due to the lack of health-coefficients. Pollutants such as PM_2.5_ and PM_10_ heavily affects long-term health. Thus, the representativeness evaluation of PM may serve as an indicator of long-term health risks. Another critical step is to enable the public to identify their best-representing FSM station by more than rough guidance based on administrative boundaries or proximity to the next station [35]. We suggest more research into effective and empowering use of FSM network data.

The representativeness of current FSM in Hong Kong is still challenging for individual pollutants, especially for local pollutants such as NO_2_. The majority of stations represent less than 1 km^2^ area for NO_2_. This is in line with previous findings from general stations in the city-centre of Paris [19]. Our results further show that most of the stations well represents less than 20,000 people. Apart from the sometimes suggested [60], but in reality arguably difficult reallocation of sensors, smart city infrastructure opens a promising avenue to increase representation of the population [61]. For example, low-cost sensors can be deployed at a multitude of locations with a relatively cheap price tag for set-up and maintenance. However, such sensors suffer from issues of drift, interference and low lifetime [9]. An integration of smart-city infrastructure into existing FSM networks is thus a challenging task. Here, the proposed network index can consider both traditional and new approaches towards measuring air quality. A higher score of the network index can be achieved both by adding high-grade traditional sensors (limited to few locations, but with a high measurement quality) and low-cost sensors (at many locations, but with more observation error). The observation error of low-cost sensors as sensor drift [62] can be accommodated by the addition of an error-term during SF-calculation. Further research is warranted to show the cost-effectiveness of both approaches, as well as their optimal locations based on an optimization for population-health representativeness.

As global cities are increasingly interwoven, data driven [63] and in competition, managing environmental pollution becomes an essential criterion for the success of a city [27]. Here, due to its grounding in health and population, the proposed metrics make management infrastructures of environmental pollution (FSM networks) comparable between cities of different sizes and characteristics. Thus, it would be of great interest to expand the proposed methodology to several of cities, consequently enabling the addition of FSM network quality as a factor of sustainability indices and city rankings.

## Conclusions

Existing methodologies judge the representativeness of urban air quality monitoring networks by spatial representativeness of a single pollutant. This paper demonstrates shortcomings of this approach. Firstly, the representativeness of multiple pollutants cannot be combined into a single metric. Secondly, we show that in Hong Kong, spatial representatives areas sizes are not correlated with actual represented population (p > 0.05 for all pollutants, n = 16). To overcome these challenges, we derived a health -and population-based metric (PHNI) for the representativeness of urban air quality monitoring networks. By basing the metric not on represented pollution concentration, but on represented additive short-term health impacts of the pollutants (%*AR*), an overarching %*AR*_*total*_ can be calculated. Results show that in our study area, representativeness values varied with pollutants, with regionally influenced PM better represented (PHNI_PM10_ = 0.87, PHNI_PM2.5_ = 0.82) than more locally gaseous pollutants (PHNI_NO2_ = 0.59, PHNI_O3_ = 0.78). Due to the dominance of these gaseous pollutants on %*AR*_*total*_ as well as their anticorrelated chemical interplay in Hong Kong’s VOC-limited regime, total short-term health effects of the large majority of the population (99.9% of 7.5 million residents) was found to be well represented by the current sensor network (SF > 0.9). In contrast to that, the well-represented population was 2.7 million, 1.6 million, 1.0 million, and 0.5 million for PM_10_, PM_2.5_, O_3_, NO_2_, respectively. District-level difference in representation quality were especially large for NO_2_, with rural districts being less-well represented than urban districts. We explain this by the relative abundance of monitors in smaller, urban districts and dedicated roadside stations capturing spatially and temporally varying traffic emissions. The adoption of a population-health based framework makes monitoring efforts comparable and can lead to improved inter-city competition for sustainable development.
